# Examining the use of alternative light sources in medico-legal assessments of blunt-force trauma: a systematic review

**DOI:** 10.1007/s00414-024-03262-8

**Published:** 2024-06-07

**Authors:** Alexander Tyr, Nina Heldring, Brita Zilg

**Affiliations:** 1 Swedish National Board of Forensic Medicine, Retzius v. 5, Stockholm, 171 65 Sweden; 2 Department of Oncology-Pathology, Karolinska Institutet, Retzius v. 3, Stockholm, 171 77 Sweden

**Keywords:** Forensic science, Bruising, Detection, Enhancing, Wavelengths, Criminology, Clinical forensic medicine

## Abstract

The ability to analyze blunt-force trauma is crucial for deciphering valuable clues concerning mechanisms of injury and as evidence for medico-legal investigations. The use of alternate light sources (ALS) has been studied over the past decade, and is proposed to outperform conventional white light (CWL) during bruise assessments. In response to the growing interest of the technology worldwide, a systematic review of the literature was conducted according to the Preferred Reporting Items for Systematic Review and Meta-Analysis (PRISMA) to address the ability of ALS to detect and visualize bruising. From an initial 4055 records identified, ten studies met the eligibly criteria and were selected for this review. Evaluation also included a novel framework, referred to as SPICOT, to further systematically assess both scientific evidence and risk of bias in forensic literature. Analysis reveals that narrowband wavelengths within in the infrared or ultraviolet spectral ranges do not significantly outperform CWL in visualizing or detecting bruising. However, wavelengths within the visible spectrum, particularly 415 nm combined with longpass or bandpass yellow filters, are more effective. However, the majority of selected studies only address the sensitivity of ALS, and therefore, results may only be considered valid when the location of a bruise is known. Further investigation is required to understand the specificity of ALS, in particular how the use of topical cosmetic products, previous wounds/scar-tissue, tattoos, moles and freckles may affect detection. The ethical concern regarding the interpretation of enhanced visualized trauma should also be considered in prospect discussions prior to implementing ALS into routine practice. Nevertheless, this review finds that narrowband ALS within the visible spectrum demonstrates potential for improved injury documentation, outperforming CWL in the detection and visualization of bruising.

**Supplementary Information:**

The online version contains supplementary material available at 10.1007/s00414-024-03262-8.

## Introduction

Bruises serve as markers of blunt-force trauma and may yield valuable clues into the mechanisms of injury [1]. An accurate and comprehensive bruise analysis is therefore warranted in cases of suspected abuse and assault. However, despite its forensic significance, the task of identifying and documenting bruises remains difficult due to a myriad of factors influencing their visibility. This includes, the degree of inflicted trauma, the dynamic and distinct process of healing [2] as well as the diversity of varying skin tones [3–6], that may result in the absence of visible bruising or the presence of bruises deem too minor to document during medico-legal examinations [7]. To overcome this challenge, a growing volume of research has explored the possibility of using alternate light sources (ALS) to enhance blunt-force trauma documentation [8].

**Fig. 1:**
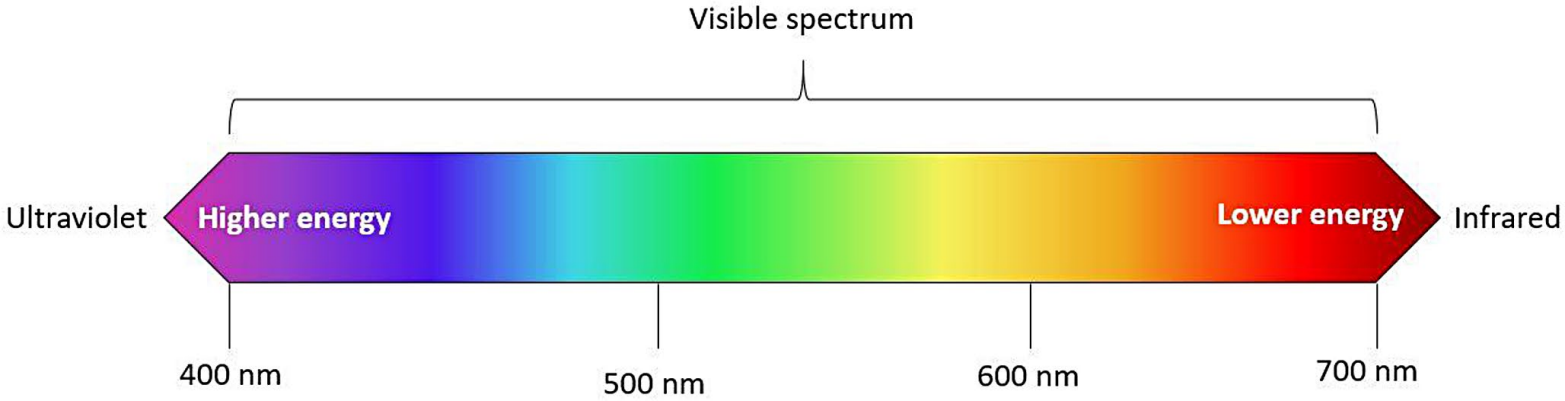
The electromagnetic spectrum. UV wavelengths, with values under 400 nm, exhibit greater energy compared to IR wavelengths, which reside above 700 nm on the spectrum’s opposite end. Longer wavelengths with lower energy can penetrate tissues more deeply than their shorter counterparts. The VLS spans from 400 nm to 700 nm, encompassing the vibrant colors of violet, blue, green, yellow, orange, and red

Light can be categorized by its wavelength into the visible light spectrum (VLS), narrowband light between 400 and 700 nm, and the invisible light spectrum, comprising both ultraviolet (UV) and infrared (IR) light composed of wavelengths below 400 nm and above 700 nm, respectively (Fig. 1). ALS refers to the use of single and narrowband wavelengths within the full spectra for illumination and are used by law-enforcement worldwide to detect biological traces such as blood and semen, as well as chemical agents including gunshot residue [9–15]. When photons of particular wavelengths are absorbed, they induce electron transitions to higher energy orbits. Fluorescence occurs when excited electrons return to lower energy states, releasing energy in the form of photons with a lower energy and longer wavelength compared to the excitation light, referred to as Stoke’s Shift [9]. Consequently, emitted light is not visible to the naked eye, requiring the use of specific longpass or bandpass filters that block the return of the excitation light [16].

The hallmark of bruising is the discoloration that occurs as a consequence of ruptured vessels in the dermal layer of the skin. Visualizing the extravasated blood using normal or conventional white light (CWL) is challenging however, as the majority of light is both reflected by the skin’s surface and absorbed by melanin, secreted by melanocytes located between the surface and dermal layer [17]. This becomes particularly prevalent in darker skin where higher concentrations of melanocytes persist. On the other hand, emission of a single or narrowband wavelength may penetrate the skin and be absorbed specifically by hemoglobin and its associated breakdown products [18, 19]. This can be perceived as darkened regions on the skin when viewed through distinct filters [20]. Hence, employing ALS to visualize bruising may circumvent the obstacles presented by white light reflection and melanin concentrations.

In the age of evidence-based medicine, forensic methods must demonstrate their scientific rigor to ensure that accurate and reliable results are presented during legal proceedings. Consequently, examining the specificity and sensitivity of ALS to understand its effectiveness in discerning bruising from non-bruising, and detecting all bruising, is paramount. Bruise detection and bruise visibility are related concepts, but refer to different aspects of bruise sensitivity. Bruise detection is the process of identifying the presence of a bruise, while bruise visibility relates to how apparent or noticeable a bruise is once it has been detected. Specificity on the other hand refers to the ability to differentiate bruising from non-bruising. In pursuit of such knowledge, we focus here answering the question: does the detection and visualization by ALS of blunt-force trauma outperform CWL approaches in medico-legal contexts?

## Methodology

### Research question

A systematic review of the literature was conducted according to the Preferred Reporting Items for Systematic Review and Meta-Analysis (PRISMA) framework [21, 22]. The objective was to address the research question: “does detection and visualization of bruising by ALS outperform CWL approaches in medico-legal contexts?”

### Search strategy and data sources

Relevant search terms were defined following consultation with an information specialist. Search queries are described in Table 1, and were constructed using the Boolean operators “AND” and “OR”. Records were collected from the databases of PubMed, Medline, and CINAHL, from inception to 30 April 2024. Supplementary sources were also extracted from citations lists of selected studies if deemed relevant.

**Table 1:** Search queries used for each database in order to extract records for screening

Set	Intervention:	Pubmed	CINAHL	Medline
**1**	**Blunt-force trauma**	Bruis*[tiab] OR contusion*[tiab] OR hematoma[tiab] OR wound*[tiab] OR non-penetrating[tiab] OR color[tiab] OR mark*[tiab] OR pigment* [tiab] OR skin[tiab] OR swelling[tiab] OR ecchymosis[tiab] OR discolor*[tiab] OR “bite mark“[tiab] OR bite[tiab] OR “blunt-force“[tiab] OR “blunt force“[tiab] OR “Contusions“[Mesh] OR “Hematoma“[Mesh] OR “Wounds and Injuries“[Mesh] OR “Ecchymosis“[Mesh] OR “Wounds, Nonpenetrating“[Mesh] OR “blunt-force trauma“[tiab] OR Blemish[tiab] OR injur*[tiab]	(TI Bruis* OR AB Bruis*) OR (TI contusion* OR AB contusion*) OR (TI hematoma OR AB hematoma) OR (TI wound* OR AB wound*) OR (TI non-penetrating OR AB non-penetrating) OR (TI color OR AB color) OR (TI mark* OR AB mark*) OR (TI pigment* OR AB pigment*) OR (TI skin OR AB skin) OR (TI swelling OR AB swelling) OR (TI ecchymosis OR AB ecchymosis) OR (TI discolor* OR AB discolor*) OR (TI “Bite mark” OR AB “Bite mark”) OR (TI Bite OR AB Bite) OR (TI blunt-force OR AB blunt-force) OR (TI “blunt force” OR AB “blunt force”) OR (MH Contusions+) OR (MH Hematoma+) OR (MH “Wounds and Injuries+”) OR (MH Ecchymosis+) OR (MH “Wounds, Nonpenetrating+”) OR (TI “blunt-force trauma” OR AB “blunt-force trauma”) OR (TI Blemish OR AB Blemish) OR (TI injur* OR AB injur*)	Bruis*.tw. OR contusion*.tw. OR hematoma.tw. OR wound*.tw. OR non-penetrating.tw. OR color.tw. OR mark*.tw. OR pigment*.tw. OR skin.tw. OR swelling.tw. OR ecchymosis.tw. OR discolor*.tw. OR “Bite mark”.tw. OR Bite.tw. OR blunt-force.tw. OR “blunt force”.tw. OR exp Contusions/ OR exp Hematoma/ OR exp “Wounds and Injuries”/ OR exp Ecchymosis/ OR exp “Wounds, Nonpenetrating”/ OR “blunt-force trauma”.tw. OR Blemish.tw. OR injur*.tw.
	Items found	4 586 646	856 942	4 524 404
**2**	**Light sources**	“alternative light sources“[tiab] OR ALS[tiab] OR “alternate light“[tiab] OR ultraviolet[tiab] OR wavelength*[tiab] OR absorption[tiab] OR UV[tiab] OR infrared[tiab] OR IR[tiab] OR narrowband[tiab] OR fluorescen*[tiab] OR “forensic light“[tiab] OR detection[tiab] OR “white light“[tiab] OR “light sources“[tiab] OR “light source“[tiab] OR “Ultraviolet Rays“[Mesh] OR “Infrared Rays“[Mesh]	(TI “alternative light sources” OR AB “alternative light sources”) OR (TI ALS OR AB ALS) OR (TI “alternate light” OR AB “alternate light”) OR (TI ultraviolet OR AB ultraviolet) OR (TI wavelength* OR AB wavelength*) OR (TI absorption OR AB absorption) OR (TI UV OR AB UV) OR (TI infrared OR AB infrared) OR (TI narrowband OR AB narrowband) OR (TI fluorescen* OR AB fluorescen*) OR (TI “forensic light” OR AB “forensic light”) OR (TI detection OR AB detection) OR (TI “white light” OR AB “white light”) OR (TI “light sources” OR AB “light sources”) OR (TI “light source” OR AB “light source”) OR (MH “Ultraviolet Rays+”) OR (MH “Infrared Rays+”)	“alternative light sources”.tw. OR ALS.tw. OR “alternate light”.tw. OR ultraviolet.tw. OR wavelength*.tw. OR absorption.tw. OR UV.tw. OR infrared.tw. OR IR.tw. OR narrowband.tw. OR fluorescen*.tw. OR “forensic light”.tw. OR detection.tw. OR “white light”.tw. OR “light sources”.tw. OR “light source”.tw. OR exp “Ultraviolet Rays”/ OR exp “Infrared Rays”/
	Items found	2 490 086	168 154	2 463 374
**3**	**Forensic medicine**	“forensic science“[tiab] OR “forensic pathology“[tiab] OR “medicolegal investigation“[tiab] OR “medico-legal“[tiab] OR judicia*[tiab] OR “forensic medicine“[tiab] OR “forensic nursing“[tiab] OR “Forensic Sciences“[Mesh] OR “Jurisprudence“[Mesh]	(TI “forensic science” OR AB “forensic science”) OR (TI “forensic pathology” OR AB “forensic pathology”) OR (TI “medicolegal investigation” OR AB “medicolegal investigation”) OR (TI medico-legal OR AB medico-legal) OR (TI judicia* OR AB judicia*) OR (TI “forensic medicine” OR AB “forensic medicine”) OR (TI “forensic nursing” OR AB “forensic nursing”) OR (MH “Forensic Sciences+”) OR (MH Jurisprudence+)	“forensic science”.tw. OR “forensic pathology”.tw. OR “medicolegal investigation”.tw. OR medico-legal.tw. OR judicia*.tw. OR “forensic medicine”.tw. OR “forensic nursing”.tw. OR exp “Forensic Sciences”/ OR exp Jurisprudence/
	Items found	335	110 591	333 149
**4**	**#1 AND #2 AND #3**	**2 587**	**337**	**2 482**
**5**	**Filter: English language**	2305	332	2 194
**6 **	**Filter: Human**	**1883**	**332**	**1 840**

### Eligibility criteria

Inclusion and exclusion criteria were defined according to the research question that defined the population, intervention, comparison and outcome (PICO). Inclusion criteria consisted of English language records published in peer-reviewed journals. Studies needed to include a sample population that was of a human model, with living individuals that presented bruising from blunt-force trauma (including bite marks). The source of the trauma was not defined. Studies needed to exhibit an intervention consisting of an ALS (UV, narrowband visible light or IR) with a CWL comparison. Records also needed to include a discussion regarding outcomes, including a statement summarizing the preferred method for visualizing or detecting a bruising. Investigations using ALS to identify biological samples outside the body such as sperm, fingerprints or gunshot reside were excluded.

### Selection of evidence

Data was imported into Microsoft Excel (Office 2019) for further selection and cataloging. Following removal of duplications, records were screened for relevance in a systematic and sequential manner, by title, abstract and full-text. Relevance of each study was assessed by two independent researchers. Disagreements were solved during consensus discussions. Only articles detailing an original study were selected for full-text screening and editorials/commentaries, conferences proceedings, case reports and technical protocols were excluded.

### Study evaluation

Studies were evaluated using SPICOT (**S**tudy design, study **p**opulation, **i**ntervention/exposure, **c**ontrols/comparisons/index test, **o**utcome and **t**imespan) to systematically assess both scientific evidence and risk of bias the forensic literature (Supplementary 1). Screening using SPICOT was conducted to ensure that only studies fulfilling established scientific criteria were selected to form conclusions in this review.

For the risk of bias assessment in SPICOT, a predetermined set of criteria within a study’s population, control/comparison, exposure and assessment were analyzed. Within the population criterion, we examined if the population had firstly been defined, secondly if bruising was controlled for or validated, and thirdly the investigated sample size. Similarly, for controls/comparison, we examined if a negative bruise assessment had been performed, and if a CWL control had been conducted, alongside identifying sample size. For intervetion, the ALS exposure had to be defined and for the assessment criterion, we examined not only if procedures had been defined, but also if multiple independent assessors were employed and if blinded assessments had occurred.

All studies were assessed in each category described to determine a combined level of evidence and risk of bias (categorized as low (0–9 points), medium (10–16 points), or high (17–20 points)). This scoring process was carried out by a sole researcher. Those scoring SPICOT-low and SPICOT-medium, were additionally assessed by a separate independent researcher. If variations in scores impacted SPICOT classification, consensus discussions were held to decide final score. Studies that both researchers identified as having a SPICOT-low were excluded.

### Data extraction

A summary of the information extracted from studies is described in Table 2. In brief, this included publication type and details regarding date of publication. The data source was also extracted in addition to an identification of the study design by the researcher. Information regarding study population was extracted, including age and skin color, as well as bruise infliction method and location on body. Population size (*n*) was also extracted. The ALS wavelength was noted alongside the specific band/longpass filter used for detection. Assessment timepoint(s) and metrics were extracted, as well as the methods used for processing of data/analysis, alongside information relating to the relevance of controls and control group size (*n*). Descriptions regarding the effectiveness in detecting and visualizing bruising using both ALS and CWL was recorded.

**Table 2:** Summary of information extracted from studies

Domain	Criterion
Publication type	Original articles
Publication reliability	Peer-reviewed publication
	Date of the publication
Data sources	Human model, antemortem / postmortem
Study design	Descriptive, correlation, causal-effect or experimental
Population/sample study	Representativeness of the population/sample (individuals with blunt-force trauma)
	Inclusion, and exclusion criteria
	Size of the population/sample
Intervention	Use of ALS (UV, VLS (400–700 nm) and IR) for detection and visualization of blunt force trauma Wavelength Longpass/bandpass filters
Controls	Relevant controls (CWL)
	Size of the control group
Outcome	Description of the outcome variable, effectiveness in detecting and visualizing blunt-force trauma injuries in medio-legal contexts

### Ethical consideration

This study involves the analysis of existing published data and therefore did not require ethical approval.

## Results

### Study selection

**Fig. 2:**
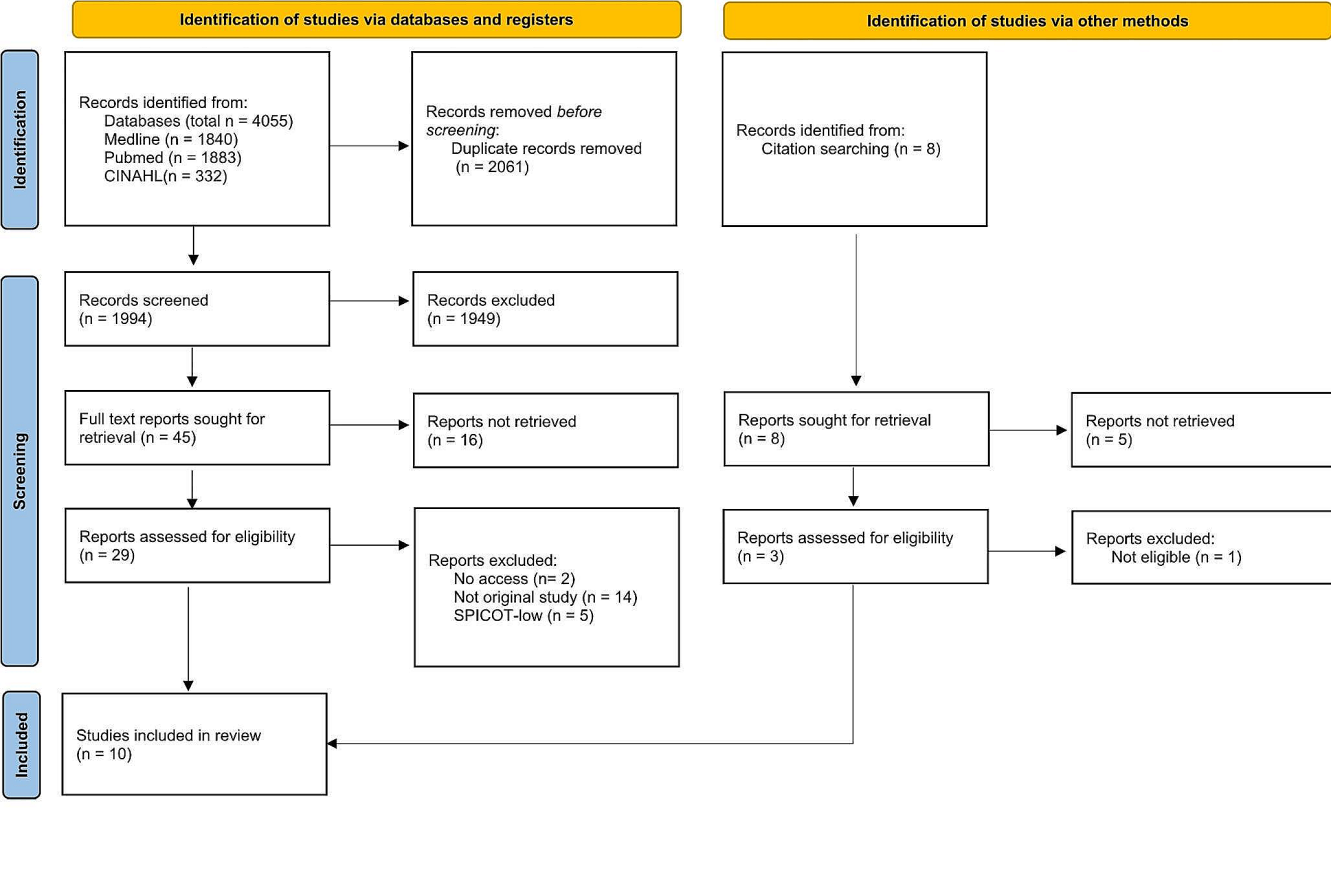
Flow chart detailing study selection

The search strategy yielded a total of 4055 studies, comprising 1883 from PubMed, 1840 from Medline and 332 from CINAHL. After removal of duplicates (2061) and systematic screening of titles and abstracts, 32 full-text articles were assessed for eligibility and 15 were further considered for SPICOT evaluation. Five studies were assessed as SPICOT-low [7, 23–26] and therefore excluded, resulting in a total of ten studies being selected for this review [6, 8, 20, 27–33]. The selection process is detailed in Fig. 2 according to PRISMA guidelines [22].

### Risk of bias assessment

Risk of bias assessment is represented in Table 3. The selected studies all had defined populations, with the majority exhibiting samples > 20 individuals. Only one study did not use an inflicted bruising control or consider a validation method to confirm bruising. While all studies conducted a CWL control, 40% did not consider a negative bruise examination/validation. In terms of assessment strategies, 60% of studies conducted blinded analysis of bruising with multiple assessors. All studies defined their ALS exposure.

**Table 3:** Risk of bias of selected studies

Reference	Population			Control/comparison			Exposure^6^	Assessment^7^
	Defined^1^	Selection^2^	Quantity^3^	Bruise comparator^4^	White light control^5^	Quantity^3^		
Limmen et al. [20]	+	+	+ +		+	+	+	+
Scafide et al. [31]	+	+	+	+	+	+	+	+
Lombardi et al. [29]	+	+	+ +	+	+	+ +	+	+ + +
Nijs et al. [30]	+	+	+ +		+	+ +	+	+ + +
Trefan et al. [27]	+		+		+	+	+	+
Scafide et al. [32]	+	+	+ +	+	+	+ +	+	+ + +
Scafide et al. [6]	+	+	+ +	+	+	+ +	+	+ + +
Black et al. [28]	+	+	+		+	+	+	+
Scafide et al. [33]	+	+	+ +	+	+	+ +	+	+ + +
Downing et al. [8]	+	+	+ +	+	+	+ +	+	+ + +

^1^Defined (+)

^2^Standardized induced bruising / validated bruise (+)

^3^Sample size is < 20 (+) and > 20 (+ +)

^4^Negative bruise examination / validated (+)

^5^White light exposure is defined/compared (+)

^6^ALS exposure is defined (+)

^7^Assessment procedure is defined (+); with multiple independent assessors (+ +) and blinded (+ + +)

### Characteristics of individual sources of evidence

Characteristics of the individual studies are summarized in Table 4. Analysis demonstrates that 10% of the studies exhibited a correlation study design, 40% had a causal-effect design, and the remaining 50% had an experimental setup. The eight studies employing a controlled inflicted bruising, consisted of either a dropped metal object onto the forearm of an individual, or by paintballs fired at the upper arm. In both cases, the velocity and impact zone were controlled. The remaining studies examined bruises within clinical settings, where timing (assessment post-trauma), bruise site (area on body) and impact details (velocity) could not be controlled for. Regarding the ALS narrowband used, the majority investigated single wavelengths within the UV and VLS, with one study exploring IR and UV wavelengths in comparison to other imaging modalities in CWL, and another study examined only IR in comparison to CWL imaging techniques. It is worth noting that only one study analyzed fluorescence while the remaining examined absorption under ALS. Diagnostic measurement was considered as: sensitivity – examination was only conducted on injuries in known locations; specificity – examination was conducted on both bruising and non-bruising sites. Based on this criterion, only one study considered specificity in their diagnostic measurement. Four studies reported bruise assessments using descriptors for visibility (e.g., clear, no, bare), two measured bruise size, one, anatomical location and another, the contrast between bruised and non-bruised skin. The remaining studies utilized a novel bruise visibility scale (BVS) and absorption visibility scale (AVS). Two studies examined bruising at a single time point, whereas the remaining eight spanned a period from 30 min post-bruise infliction to four weeks post-bruising. Two studies did not report or consider their sample population skin color, with half of the remaining eight exhibiting representation across six skin categories: “very light,” “light,” “intermediate,” “tan,” “brown,” and “dark”. The remaining 50% had predominantly “white”/“light” sample populations.

**Table 4:** Characteristics of studies examining bruise detection and visualization using ALS compared to CWL

Reference	Aim	Study design	Bruising method	Bruise location	ALS	*n*	Diagnostic measurement	Assessment type	Bruise assessment	Assessment timepoints	Sample population skin color
Limmen et al. [20]	To assess the effectiveness of narrow-banded visible light in enhancing the visibility of injuries	Causal-effect	Uncontrolled blunt-force trauma	-	4 bandwidths within the VLS and 4 longpass filters	53	Sensitivity	Live	Visibility	Single contact. Average age of bruise was 2.6 days	-
Trefan et al. [27]	To compare bruise assessments using conventional, cross polarized, IR and UV imaging	Correlation	Uncontrolled blunt-force trauma	Leg and arm	IR, UV	25	Sensitivity	Photograph	Measurement of bruise diameter	Single contact	-
Black et al. [28]	To compare colour, cross polarised and IR imaging during bruise formation	Causal-effect	Controlled paintball strike	Leg	IR	18	Sensitivity	Photograph	Measurement of contrast by bruise area (bruising vs. non-bruising skin)	Alternate days post impact for 3 weeks	83% white, and the remaining being Arab, Asian or Black
Lombardi et al. [29]	To evaluate the performance of ALS in the detection of subclinical bruising	Experimental	Controlled trauma dropped weight	Forearm	7 single VLS wavelengths 3 filters*	118	Specificity and sensitivity	Live	Distance from the most distal antecubital fossa crease and from the most proximal wrist crease of lesion	Day 0, 1, 7, 14.	Subjects self-reported race: non- Hispanic white 66.1%, Hispanic 15.3%, Asian 11.9% and black 5.1%
Nijs et al. [30]	To study the visibility of standardized inflicted bruises by ALS compared to CWL	Causal-effect	Controlled trauma dropped weight	Forearm	Single VLS wavelength single filter*	76	Sensitivity	Photograph	Visibility	Day 0.25, 1, 2, 7, 14.	Light
Scafide et al. [31]	To compare bruise detection using ALS and CWL on a diverse sample with identical injuries	Causal-effect	Controlled trauma dropped weight	Forearm	UV and 13 single VLS wavelengths and 4 longpass filters	8	Sensitivity	Live	Visibility	30 min post-trauma, then 3–4 times per day for 3 days	3 subjects had light skin tone, 5 had a dark complexion
Scafide et al. [32]	To determine if an ALS is more effective than CWL at detecting bruises induced on diverse skin tones	Experimental	Controlled trauma dropped weight (forearm) and controlled paintball strike	Forearm and upper arm	UV and 6 single VLS wavelengths and 4 bandpass filters	156	Sensitivity	Live	Visibility	21 times over a 4-week period	Equal representation across six skin categories: very light, light, intermediate, tan, brown, dark.
Scafide et al. [33]	To evaluate detection and visibility assessments using the BVS and AVS instruments for potential future application in clinical forensic practice	Experimental (Results synthesized from parent study Scafide et al. [37])	Controlled paintball strike	Upper arm	UV and 9 single VLS wavelengths and 4 filters*	69	Sensitivity	Live	Measured bruise size (or area of absorption), contrast, and color difference	21 times over a 4-week period	Six skin categories: very light, light, intermediate, tan, brown, dark
Scafide et al. [6]	To determine which ALS combination provides the greatest predictive probability of detecting bruising on individuals with different skin tones	Experimental (Results synthesized from parent study Scafide et al. [37])	Controlled paintball strike	Upper arm	UV and 9 single VLS wavelengths, and 4 filters*	157	Sensitivity	Live	BVS and AVS	21 times over a 4-week period	Equal representation across six skin categories: very light, light, intermediate, tan, brown, dark
Downing et al. [8]	To assess if ALS enhances bruise visibility over CWL using validated tools, and explore factors that might enhance bruise visibility with ALS	Experimental (Results synthesized from parent study Scafide et al. [37])	Controlled Paintball strike	Upper arm	UV and 6 single VLS wavelengths and 4 filters*	156	Sensitivity	Live	BVS and AVS	21 times over a 4-week period	Six skin categories: very light, light, intermediate, tan, brown, dark

- not described

* longpass or bandpass not defined

### Results of individual sources of evidence

Table 5 summarizes findings presented in the selected studies. Collectively, the data indicate that among the ten selected studies, eight suggest that ALS is more effective than CWL in detecting and visualizing bruising, particularity mentioning its usefulness during early stages of bruise formation. Analysis reveals that wavelength filter combinations within the IR or UV spectral ranges do not outperform CWL, while narrowband wavelengths within the VLS, specifically 415 nm combined with either longpass or bandpass yellow-cut filters do.

**Table 5:** Reported results and conclusions of studies examining bruise detection and visualization using ALS compared to CWL

Reference	Main findings reported	Most effective wavelengths (nm)	Study conclusions	Examination method supported
Limmen et al. [20]	Approximately 43% of all examined injuries showed an improvement in visibility when exposed to a crime-lite®.	400–430 and 430–470 using corresponding longpass filters	Crime-lites®, 400–430 violet and 430–470 blue enhance visibility of bruises barely visible in CWL. Crime-lites® enable the visualization of injuries that would otherwise remain invisible or go unnoticed.	ALS
Trefan et al. [27]	IR and UV imaging techniques were not better at visualizing bruises compared to conventional and cross polarized methods. Cross polarized (CP) and UV images provide sizes similar to that seen in a conventional imaging, but IR results in a smaller measurement.	-	It is possible to define the size of a bruise across imaging modalities, as no difference between methods were noted.	CWL
Black et al. [28]	There was no significant difference between photographic techniques when a bruise was visible in CWL. IR imaging resulted in a greater impact mark compared to colour and CP methods immediately post-trauma.	-	IR was marginally better at visualizing subcutaneous bleeding than color and CP imaging in CWL at the early stages of bruising, though to no significant degree.	ALS during early stages of bruising
Lombardi et al. [29]	Average sensitivity reported on day 1 was 76.8%, that dropped to 69.6% on day 7 and 60.7% day 14. Average specificity day 1 was 51.6%, 59.7% day 7 and 53.2% day 14. 535 nm with a yellow filter demonstrated the highest specificity on day 1, 7 and 14 at 90.3%, 98.4% and 96.8% though sensitivity was 19.6, 8.9 and 3.6 days 1, 7 and 14, respectively. 415 nm with yellow filter resulted in a specificity of 51.6% day 1, 62.9% day 7 and 53.2% day 14.	535 with yellow filter*	Bruise detection under CWL decreases over time, while the ALS maintained consistently high sensitivity in detecting inflicted bruises. 14 days post-trauma, the ALS identified nearly twice as many subjects with inflicted trauma compared to CWL. However, CWL retained greater specificity, distinguishing false positives more effectively than ALS.	CWL
Nijs et al. [30]	Most bruises were visible both with an ALS and CWL. The score ‘no visible bruise’ was given more often with an ALS than with a CWL. Mean report marks for bruise visibility with an ALS compared to a CWL were significantly higher at 1 and 2 days after impact. Other time points exhibited no significant differences.	Only 415 with yellow filter* combination tested	Limited value of ALS for enhancing bruise visibility immediately and following 2 days post injury	ALS during early stages of bruising (CWL better immediately)
Scafide et al. [31]	Bruising was detectable in 78% of assessments. Of the assessments where bruising was detected, 98% were detected by the ALS while 24% were by CWL. 34% of the total bruises not detectable under CWL were visible under ALS assessments.	Wavelengths (415–450). No association was noted with different longpass filters	Although CWL was more effective in visualizing bruises closer in time to bruise creation, ALS detected bruises more consistently over the first 3-day period. Thus, ALS has a greater likelihood of detecting faint bruises during the first three days post injury.	ALS during early stages of bruising (CWL better immediately)
Scafide et al. [32]	More bruises were visible under ALS than CWL (81.8 vs. 50.8%, respectively) over the 4-week period. The paintball mechanism resulted in visible bruises in all participants under CWL at the first assessment (30 min post-infliction). Bruising was most frequently observed under 415–450 nm with a yellow filter and had greater odds of detecting a bruise than CWL. All other wavelength had lower odds of detecting bruises compared to CWL.	415 and 450 with yellow longpass filter combinations	Absorption was detected under ALS more frequently than visible discoloration under CWL for both upper and lower arms.	ALS
Scafide et al. [33]	Interrater aggreement was over 90% for all assessments, except for wavelengths 515 and 535 nm with the red filters. Size of the bruise (area of absorption) was significantly associated with visibility score for both ALS and CWL, and the degree of contrast between the bruise and surrounding skin is an indicator of bruise clarity.	415 with yellow filter*	BVS and AVS are reliable and valid measures of bruise visibility when under CWL and ALS, respectively.	ALS using a AVS and CWL using a BVS
Scafide et al. [6]	Among all six skin pigmentation categories, ALS wavelengths of 415 nm and 450 nm and (yellow filter), exhibited the highest frequency of bruise detections (415 nm 11.2%; 450 nm 11.1%) and demonstrated a higher likelihood of bruising compared to CWL.	415 and 450 wavelengths with yellow filter* combination	415 and 450 nm with yellow filter were the only wavelengths better than CWL in detecting bruising in individuals with darker skin tones (brown or dark).	ALS
Downing et al. [8]	The frequency of visible observations decreased with increasing skin pigmentation: very light 19.5%, light 20.7%, intermediate 18.1%, tan 16.0%, brown 16.2%, and dark skin 9.6%. Only 415 nm viewed through a yellow filter resulted in a clinically meaningful improvement in visibility rating when compared to CWL.	415 nm with yellow filter*	415 with yellow filter better than CWL at determine bruise visibility using AVS. UV was inferior compared to CWL, particularly on individuals with dark skin pigmentation.	ALS

- not described

* longpass or bandpass not defined

Various studies [6, 8, 29, 32, 33] have explored the effectiveness of different single wavelength and filter combinations in detecting and enhancing bruise visibility compared to CWL. Limmen et al. [20] demonstrated that narrowband wavelengths between 400 and 470 nm significantly increased visibility compared to CWL, reporting an improved visibility in 52% of bruises that were initially deemed “barely visible” under CWL. These finding are consistent with the known absorption peaks of oxyhemoglobin (415 nm), de-oxygemoglobin (430 nm), and bilirubin (460 nm) [18, 19, 34]. Despite the declining frequency of visible observations with increasing skin pigmentation [8], wavelengths of 415 nm and 450 nm (paired with a yellow filter) exhibited the highest rates of bruise detection across all skin categories (415 nm: 11.2%; 450 nm: 11.1%), with 415 nm/yellow filter being the only combination that outperformed CWL in cases where skin colour was classed as “brown” or “dark” [6].

Although the ability to detect bruises decreases over time, results from the selected literature implies that bruising may be detected and visualized sooner following trauma with an ALS than with CWL [28–31]. Scafide et al. [31] identified bruising in 98% of cases within the initial three days post-trauma when employing 415 nm /yellow filter combination, whereas only 24% were detectable under CWL. Although the use of IR was proposed to be marginally superior to CWL during bruise formation in Black et al. [28], no statistically significant difference was observed between the methods. Findings are similar to that reported by Trefan et al. [27], though IR imaging was noted to produce smaller bruise sizes compared to CWL imaging.

The time frame for when ALS is more effective than CWL appears to be constrained at both ends, as studies suggest that CWL is better within the initial 30 min post-trauma [30, 31] and at earliest after two days post-trauma [30]. Though further investigations are needed, as findings reported are contrasting. For instance, Nijs et al. [30] found no significance between bruise visibility under ALS and CWL seven days post-trauma using 415 nm/yellow filter combinations while findings by Scafide et al. [32] noted that the 450 nm /yellow filter consistently outperformed CWL in detecting bruises within a 4 week period post-injury. However, differences in analysis may account for these differences as Nijs et al. [30] examined bruise visibility and Scafide et al. [32] bruise detection. Nevertheless, the proposed time frame may explain why ALS performed better than CWL in the study by Limmen et al. [20], where the average time between injury and ALS examination was 2.6 days.

The quantification of the visual degree of bruising conducted initially by Nijs et al. [30]. expressed between one (very bad) and ten (excellent), circumvents subjective visibility descriptors such as “obvious,” “clear,” “distinct,” “faded,” and “faint”. Scafide et al. [33] further developed this quantitative BVS, and suggesting that visibility should not be measured using the same scale for both CWL and ALS, since CWL includes the entire VLS and ALS only a narrow bandwidth. This may explain why bruises of low contrast, i.e. difficult to distinguish from surrounding skin, are more diffuse and less distinctive using IR and UV light [27]. Scafide et al. [33] therefore proposed a tailored BVS, referred to as the AVS when using ALS. When scales were compared, a greater bruise size was associated with higher visibility using either scale but that greater contrast in color or lightness was associated with higher BVS values alone [33]. Future studies should therefore consider the use of the AVS to provide more unity between investigations and comparable results.

## Discussion

Unlike traditional forensic medicine that often relies on singular observations during autopsies, research within clinical forensic medicine benefits from being able to employ experimental study designs akin to those used in clinical trials. For instance, the majority of research investigating the effectiveness of ALS compared to CWL, involve randomized study populations, controlled bruise inflictions, and examination strategies using multiple contact points with blinded assessments.

From an initial search encompassing 4055 records, ten articles were identified to meet the specified inclusion and exclusion criteria post screening. Data extracted from the selected studies indicate that employing a 415 nm ALS combined with a yellow bandpass/longpass filter outperforms CWL in both bruise detection and visualization. While research in this area is restricted to a single study, findings demonstrate that the 415 nm/yellow filter combination also performs better than CWL and other narrowband wavelengths when assessing bruises in individuals with darker skin tones. However, this is provided the location of a trauma is known. Only a single study compared the ability of ALS to discern bruising from non-bruising, with results indicating that caution is warranted if examining fluorescence [29].

Previous studies have raised concerns regarding the specificity of ALS in detecting bruising [29, 35, 36]. The chart review by Holbrook and Jackson [7] showcased an impressive capability of ALS to detect bruises, identifying bruising in 98% of reported cases of strangulation, wherein 93% displayed no apparent injuries under CWL examination. This highlighted the use of ALS as a compelling tool for bruise detection, with the findings presented in legal proceedings [29]. However, the absence of controls specifically addressing bruise validity limits the results [7], as ascertaining what the authors’ identified as bruising is perplexing, since neither hemoglobin nor bilirubin exhibit significant fluorescent properties, and skin may fluorescence from factors other than bruising [17, 37]. Further investigations by Lombardi et al. [29] revealed that a CWL had a significantly greater specificity compared to fluorescence under ALS. Authors concluded that the diagnostic reliability of fluorescence under ALS remains uncertain if bruising cannot be validated, and further investigation examining the specificity of absorption is necessary. Debatably, Lombardi et al. [29] presentation of results by pooling wavelengths into a single sensitivity and specificity measure may be deemed inaccurate, as data from individual wavelengths do indeed exhibit higher sensitivity and specificity than CWL at various time points during the course of the experiment. Nevertheless, to alleviate problems associated with the lack of specificity in routine casework, ALS examinations should always be conducted in conjunction with CWL. This approach facilitates the evaluation of additional factors including pain, swelling, and the patient’s history of physical trauma to validate bruising.

Moreover, common over-the-counter topical products have demonstrated to generate greater ALS absorption when applied on light or medium skin tones compared to those with dark skin [37]. One makeup product consistently absorbed wavelengths between 310 and 535 nm in 80.9% of observations, and sunscreen (SPF30) absorbed significant light in 7% of cases. However, the remaining twelve products tested absorbed light in less than 1% of observations [37]. In a follow up study evaluating the effectiveness of three different topical product removal methods (soap and water, isopropyl alcohol swab, makeup removal wipe), four out of 14 products continued to exhibit significant absorption after removal [38]. No differences were noted between removal methods, highlighting that further research exploring the specificity of ALS and topical products post-inflicted trauma is warranted, alongside studies questions relating to how previous wounds/scar-tissue, tattoos, moles (including Mongolian spots) and freckles affect specificity. Thus, live ALS examination is therefore advocated to ensure suspected bruises can be washed to mitigate any unknown risk of interference [17]. Relying solely on ALS and CWL photography for bruise examination may overlook such elements.

Research on the ability of ALS to detect and visualize bruising across varying skin pigmentations is sparse. Although Lombardi et al. [29] disclosed that subjects were recruited regardless of race, only a small fraction exhibited dark skin pigmentation. The majority of the selected studies examined white/light populations. Of the ten studies reviewed, only the study series by Scafide et al. [6, 8, 32, 33] has addressed equal representation across skin categories determined by spectrophotometry. Scafide et al. [6] found that the wavelengths 415 nm and 450 nm, when paired with yellow-cut filters, were consistently better than other wavelengths at bruise detection for all tested skin categories. UV was less effective than CWL in identifying bruising across darker skin tones, except in individuals with very light skin, which may be due to melanin’s peak absorption wavelength around 335 nm [39, 40]. On the other hand, hemoglobin’s absorption spectra typically exhibits a sharp peak at around 415 nm (dependent on oxygenation level) and most probably accounts for why the wavelength was most effective [19]. Although Scafide et al. [32] initially advocated the use of yellow or orange filters, subsequent analysis using the developed AVS [33], determined that yellow alone was more effective [6]. Although results are in contrast to findings by Sully et al. [41] who suggest that longer wavelengths combined with orange filters are superior in dark skin, the use of a goat model with topically applied melanin could have resulted in higher pigment concentrations than that of human skin and may account for differences observed. Additional studies are needed for further confirmation.

Furthermore, it should be noted that all ten studies examined bruising on extremities. The location of injury has demonstrated to have a significant impact on bruising manifestation and by extension, detection and visibility. For example, the presence of loose subcutaneous tissues increases the risk of blood extravasation, leading to more pronounced bruising around specific regions such as the eye compared to the hand [1]. Subpopulations such as children and the elderly are more susceptible to bruising than young and physically fit individuals [34]. Additionally, individuals with conditions like hypertension, diabetes, and coagulation disorders are also more prone to exhibit different bruising patterns. Certain steroids have been observed to affect the rate of bruising development [42], and common medications such as anticoagulants can influence both the formation and resolution of bruises, which can manifest immediately, or take longer to develop [1, 34]. Hence, results from the selected studies are constrained by the possibility that the data may not extend to injuries sustained on the torso, face/neck, and genital regions. In practice, medical history may not always be considered prior an ALS assessment, and further studies are warranted to address such injury mechanisms and locations.

While ALS research has primarily focused on assessing the technology’s capacity to detect and visualize bruising for enhanced documentation of blunt-force trauma for legal purposes, an ethical dilemma emerges regarding a potential for overinterpretation of injury mechanisms. Although this discussion falls beyond the scope of this review, it warrants attention for future research to contemplate how enhanced visualization of bruises could inadvertently mislead legal professionals lacking medical and technical expertise. For instance, an increased visualization could result in an overestimation of injury severity or mechanism of injury, leading to erroneous judgments and unjust outcomes in legal proceedings. Hence, forensic and legal experts must exercise caution and thoroughness when interpreting and communicating ALS bruising evidence, particularly if relying solely on photographs.

### Limitations of study

This review faces several limitations stemming from predetermined constraints dictated by the nature of systematic reviews and the narrow research question. While studies examining both specificity and sensitivity were included, the strict criteria resulted in a restricted pool of eligible studies. Consequently, only ten studies were deemed suitable, with only a single addressing specificity. This selection bias should be considered when interpreting the review’s outcomes, as while ALS outperforms CWL in bruise detection and visualization, studies have only considered the technology where bruise location is known. In cases where a bruise cannot be validated either by CWL or other methods, ALS should be used with caution, as studies do not sufficiently address specificity.

It should also be mentioned that five out of the ten selected studies were authored by the same research team, four of which were derived from the same primary dataset. Such pseudoreplication of findings, albeit presented from varying perspectives, may be argued to pose a limitation to this review and the wider research domain.

## Conclusions

Conclusively, results from this systematic review indicate that ALS is more effective than CWL in detecting and visualizing bruising. Analysis reveals that wavelength filter combinations within the IR or UV spectral ranges do not outperform CWL, while wavelengths within the VLS, specifically 415 nm with either long/bandpass yellow filters do, across differing categories of skin color. These results however, only address the sensitivity of ALS, and can only be considered valid when the location of a bruise is known.

Although only a limited number of studies exist, most employ experimental designs that deliver high-quality data due to their randomization and controlled bruise infliction processes. Further investigations of comparable rigor are imperative, ideally conducted by a greater diversity of research teams. These studies should delve into questions concerning specificity, encompassing the impacts of topical products, a range of injury mechanisms, and repercussions on different anatomical regions. Moreover, the ethical quandary surrounding potential pitfalls stemming from the overinterpretation of visually enhanced data will demand careful consideration in the future, particularly as digital imaging methods become more autonomic.

### Electronic supplementary material

Below is the link to the electronic supplementary material.

Supplementary Material 1
