# Supplementary material for: Examining the use of alternative light sources in medico-legal assessments of blunt-force trauma: a systematic review
Source: Int J Legal Med. 2024 Jun 7;138(5):1925–38. doi: 10.1007/s00414-024-03262-8 (PMC11306313; doi:10.1007/s00414-024-03262-8)
Supplement: Supplementary file 1 — Supplementary Material 1 [file 414_2024_3262_MOESM1_ESM.docx]

**SPICOT Evaluation and scoring system**

SPICOT is novel framework developed to systematically evaluate forensic literature. SPICOT stands for **S**tudy design, study **p**opulation, **i**ntervention/exposure, **c**ontrols/comparisons/index test, **o**utcome and **t**imespan. All studies are attributed a score within each area that is then used to specify the evidence level as a product of the risk for bias in each article (low, medium or high), by a single researcher. In studies scoring with a high risk for bias, an independent assessment by another researcher should be conducted for corroboration. Consensus discussions should occur in cases where differences in scores alter risk categories.

1. **Study type:** Only original studies are relevant for assessment.

**1p A descriptive study** that systematically describes variables but is not based on an initial hypothesis.

**2p Correlation studies** examine the association/correlation/relationship between two or more variables to identify trends and patterns. A research question must be defined.

**4p Causal-effect studies** examine how an independent variable affects other variables with a clear research question. A comparison between two existing populations/groups must occur, where one is a control group. The research question must be defined.

**6p Experimental studies** are causal-effect studies where manipulation of **one** variable and the measurement of the other variables are evident. Randomized populations/study groups are used. The research question and hypothesis must be defined.

1. **Population/problem:** Always assessed and applicable only to the study group.

Is the population/problem described accurately in order to answer the research question? (Both criteria are met).

**0p** No

**1p** Partly

**2p** Yes

**4p**  Yes, and the study population is n ≥ 20

1. **Intervention/exposure:** Mostly exposure in forensic studies. Usually linked to the definition of the study population. If important for the study’s research question, the question would be:

Is the intervention/exposure described in order to answer the research question? (Both must be fulfilled).

**0p**  No

**1p** Partly

**2p** Yes, or not relevant

1. **Comparison/control/comparator/index test:** Mostly comparison/control in forensic studies. If important for the study’s research question, this question only applies to the control group(s) or comparison.

If a control(s) exists, is it **described and appropriate** in order to answer the research question? (Both criteria are met)

**0p** No

**1p** Partly

**2p** Yes

**4p**  Yes, and control group(s) n ≥ 20

1. **Outcome:** Do the study’s results answer the research question.

**0p** No

**1p** Partly

**2p** Yes

1. **Timespan:** Has method development occurred in the field during the study period, and has it been considered? Assess whether the problem is significant and likely to have occurred

**0p** Consideration has not been taken but is deemed necessary.

**2p** Consideration has been taken or is not necessary.

**SPICOT scoring system**

**High risk for bias** has a low total score: **1-9p**

**Medium risk for bias** has a medium total score: **10-16p**

**Low risk for bias** has a high total score: **17-20p**

Max total score is: 20p.

Min total score is: 1p.

The scoring system is designed to restrict scoring totals for both descriptive and correlational studies to no higher than a medium risk for bias, as the study design inherently delivers the lowest value of scientific evidence.

Concurrently, while causal-effect and experimental study designs have a higher evidence value due to their methodology, they are able to score high for risk of bias.
